# The Influence of Work Status Preference, Health, and Gender on Informal Caregiving Status

**DOI:** 10.1093/geroni/igaa057.108

**Published:** 2020-12-16

**Authors:** Shanika Koreshi

**Affiliations:** Massey University, Palmerston North, Manawatu-Wanganui, New Zealand

## Abstract

Current, literature has found a negative relationship between caregiving responsibilities and paid employment. There are two possible causal explanations; people with low employment prospects self-select into care and caregiving is time-consuming therefore, it require carers to accommodate this by reducing work. While there is considerable work on the influences of caregiving on work, there is limited research investigating how employment influences informal caregiving decisions. We investigated longitudinal associations between work status preferences (over-employed, underemployed, satisfied) and informal caregiving among older adults in New Zealand. The present study also explored whether gender and perceived health moderated the relationship between work status preference and caregiving. The study used data provided by three waves of the New Zealand Health, Work and Retirement Study. The findings suggest that underemployed and overemployed participants were more prone to taking up caregiving compared to participants who were satisfied with their work status. Regression analyses revealed that females with poor self-reported health were most vulnerable to experience incongruent work status preferences. The direct effects of work status preference on willingness to care may imply conflict between policies promoting full time labour force participation and social welfare policies that are continuing to rely on family carers to support the community. This evidence has implications for care policy given the importance of informal care in sustaining ageing in place policies.

